# Using brachial-ankle pulse wave velocity to screen for metabolic syndrome in community populations

**DOI:** 10.1038/srep09438

**Published:** 2015-03-30

**Authors:** Guanghua Wang, Liang Zheng, Xiankai Li, Juanli Wu, Lijuan Zhang, Jie Zhang, Liling Zou, Xin Li, Yi Zhang, Qian Zhou, Huimin Fan, Yang Li, Jue Li

**Affiliations:** 1 Shanghai First Maternity and Infant Hospital, Tongji University School of Medicine, Shanghai, 201204, China; 2 Research Center for Translational Medicine, Shanghai East Hospital, Tongji University School of Medicine, 200120, China; 3 Department of Cardiology, Shanghai Tenth People's Hospital, Tongji University School of Medicine, Shanghai, 200072, China; 4 Department of Prevention, Tongji University School of Medicine, Shanghai, 200092, China; 5 Department of Health Management Medicine, Shanghai East Hospital, Tongji University School of Medicine, Shanghai, 200120, China

## Abstract

The aim of this study is to investigate the viability of using brachial-ankle pulse wave velocity (baPWV) as a primary tool to screen metabolic syndrome (MetS), and to explore the risk factors of MetS in community populations. A total of 1914 subjects completed medical examination in Shanghai. BaPWV was significantly associated with the components of MetS. The area under curve (AUC) and its 95% confidence interval (CI) in total group were 62.50% and 60.00%–65.30% with the appropriate cut-off point being 1435 cm/sec. The AUC (95%CI) of three subgroups (40–50 yrs, 50–60 yrs and over 60 yrs group) were 75.30% (67.48%–83.35%), 63.35% (58.96%–67.60%), 55.37% (51.19%–60.01%), respectively. A clear pattern surfaced in the process of investigation: the younger were the subjects group, the better receiver operating characteristic (ROC) efficacy would emerge; and the higher sensitivity was, the better negative predictive value (NPV) would be. Male gender, high baPWV values, elevated uric acid (UA) and excess hypersensitive C reaction protein (hs-CRP) levels were stayed in the two regression models as the independent risk factors for MetS. We conclude that baPWV may serve as a potential screening tool for MetS at the cut-off point of 1435 cm/sec.

Brachial-ankle pulse wave velocity (baPWV) is a promising yet relatively simple test that measures the stiffness of both aortic and peripheral arteries^1^,^2^ and a number of research groups propose using this technique to detect subclinical atherosclerotic changes. Many studies have demonstrated its reproducibility and validity^3^,^4^,^5^, with some investigators advocating that baPWV is not only an ideal marker for vascular damage^6^,^7^ but also a valuable predictor to the mortality resulted from cardiovascular diseases^8^. Due to the non-invasive nature and simplicity in performing all the measurements, baPWV has a potential screening value for vascular injuries in community populations.

MetS involves a combination of factors that increases the risk of cardiovascular diseases and type 2 diabetes, and its prevalence is increasing in many countries and in China alike. These risk factors include obesity, dysglycemia, raised blood pressure (BP), elevated triglyceride (TG) level, and low high-density lipoprotein cholesterol (HDL-C) level^9^. With the increasing prevalence of MetS in China, MetS has seriously burdened the society and, in particular, the families of those affected subjects as reported in literature^10^,^11^.

Early detection and treatment of MetS have been proven to reduce further cardiovascular disease morbidity and mortality^12^. However, with no immediate physical symptoms or medical problems associated with this disorder, the diagnosis of MetS remains challenging. The most widely recognized metabolic criteria (lipid profile, BP and glucose level) are commonly measured during hospitalization of patients^13^. The cost and availability of these diagnostic tests have hampered the mass screening in high-risk populations using above indices, especially in underdeveloped areas and developing countries. Hence, we launched this study to investigate the feasibility of using baPWV in screening for MetS in community populations. The current study focused on the efficacy of a non-invasive baPWV examination in screening individuals at high risk of MetS. Since it is well known that the incidence of MetS increases with age, we explored the screening capability of the method and appropriate cut-off point.

## Methods

### Study population

Methods used in the present study were carried out in accordance with the approved guideline. At the same time, all experimental protocols were approved by the Ethics Committee of Tongji University, and all the participants signed informed consent.

Individuals were included if they met all the following criteria: (1) subjects with age 40 years and above; (2) subjects who were conventional residents in Lujiazui community, Shanghai; (3) individuals without hypertension crisis, multiple system organ failure, or other disease that may prevent them from measuring baPWV. Subjects were excluded if they did not meet above criteria. This community-based study adopted a cluster sampling scheme and randomly recruited a total of 2000 individuals between June 2009 and January 2010. Among these participants, 1914 subjects completed the lab tests and personal health and medical questionnaires. The response rate was 95.70%.

### Anthropometric index and lab tests

Body mass index (BMI) was calculated as body weight in kilograms divided by the square of the body height in meters (kg/m^2^). The systolic and diastolic blood pressures (SBP and DBP, respectively) were obtained from the left arms of seated patients with an automatic BP monitor after 20 minutes of rest. Blood was drawn from the subjects in the morning after a 12-hour overnight fasting and was sent for analysis within four hours of collection. Biochemical markers such as uric acid (UA), total cholesterol (TC), TG, brain natriuretic peptide (BNP), hypersensitive C reaction protein (hs-CRP), homocyteine (Hcy), HDL-C, low density lipoprotein cholesterol (LDL-C), glycosylated hemoglobin Alc (HbAlc), and fasting plasma glucose (FPG) levels were analyzed using a biochemical auto analyzer in the Department of Clinical Laboratory, Shanghai East Hospital, Tongji University School of Medicine.

### Lifestyle behaviors

In the present study, the term ‘not married’ referred to both unmarried and divorced individuals, ‘low education level’ meant less-than-12 years of full time education, and ‘high education level’ denoted equal to or greater than 12 years education. The subjects' current smoking status was derived from affirmative responses to the question “Are you a regular smoker now?” For participants reporting a history of smoking, those who had smoked within the past 6 months were defined as ‘current smokers’; while those who had not smoked within the past 6 months as ‘non-smokers’^14^. Participants were also asked “How many alcoholic drinks do you have each week?” and “How many days each week do you usually drink alcohol?” These variables were used to determine the approximate number of alcoholic drinks consumed each day by the participant. A binary variable was constructed to distinguish between the participants consuming less than two and those drinking at least two alcoholic drinks every day.

### Definition of MetS

The definition^15^,^16^ of MetS was as following:(1) Required criteria: BMI ≥ 25 kg/m^2^ (waist circumference was not available in this study); (2) Plus any two of the following four factors: a. Elevated TG level: ≥1.7 mmol/L (or 150 mg/dL); b. Reduced HDL-C level: <40 mg/dL (male) or 50 mg/dL (female); c. Raised BP: SBP ≥ 130 mmHg or DBP ≥ 85 mmHg, d. Elevated FPG level: ≥5.6 mmol/L (or 100 mg/dL).

### BaPWV measurement

A clinical device (baPWV-form) was used to automatically and simultaneously chronicle BP in both arms and ankles and document waves of the brachial and posterior tibial arteries using an automated oscillometric method^17^,^18^,^19^. BaPWV value can easily be checked through VP-1000 automated PWV/ABI analyzer (model VP-1000; Nippon Colin Ltd., Komaki, Japan). These values were measured after individuals had rested for at least 5 minutes. In case the values of baPWV obtained on the right and left sides were not identical^20^, we adopted the average value of left and right baPWV.

### Multivariate model

The adjusted variables included in Model 1 were baPWV, gender, educational level, income level, marriage status, UA, Hs-CRP and BNP, as included in the Model 2 were baPWV, gender, educational level, income level, marriage status, smoking, alcohol drinking, UA, hs-CRP and BNP.

### Statistical analysis

Continuous data were expressed as mean and standard deviation (SD). For the continuous variables, *t*-test was employed to analyze the difference between MetS and non-MetS groups. If the variances were not equal between two groups, nonparametric test was used instead of *t*-test. For the classified variables, a Chi-squared test (*χ*^2^-test) was conducted to show the ratio difference between the two groups. Receiver operating characteristic (ROC) curve^21^,^22^ was constructed to determine the optimal baPWV threshold in order to discriminate MetS and non-MetS individuals. Afterwards, the baPWV value with the highest sum of sensitivity and specificity was identified as the cut-off point. A step-wise logistic regression analysis was conducted to explore the risk factors of MetS. All statistical analyses were performed using the SPSS17.0 (SPSS Inc., Chicago, IL, USA) software package. A two-tailed *P* value of <0.05 was considered statistically significant.

## Results

### Demographic characteristics and correlation between baPWV and the components of MetS

The ages of all subjects traverse from 40 to 89 years with mean age being 59.46 ± 8.88 years. As shown in Table 1, the subjects in MetS group exhibit higher mean age and BMI compared to those in non-MetS group (*P* < 0.01). Other statistically significant differences have not been detected between the two groups of subjects, excepting the diabetes history and alcohol drinking status.

Our results demonstrate that there are significant differences between the two groups of subjects with nearly all the measured biochemical characteristics, excluding BNP, Hcy and augmentation index (AI) (Table 2). The subjects in the non-MetS group display a significantly higher ankle-brachial index (ABI) than those in the MetS group (*t* = 4.87, *P* < 0.01).

**Figure 1:**
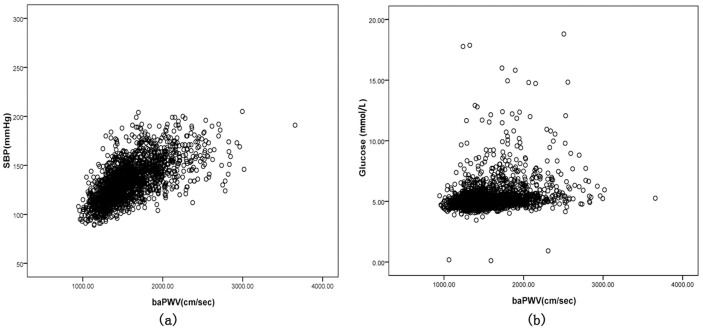
Simple linear correlation between baPWV and other variables. (a) r = 0.60, *P* = 0.001; (b) r = 0.24, *P* = 0.001.

Conspicuously, the results in Table 3 prove a negative correlation between baPWV and HDL-C (*r* = −0.09, *P* < 0.01) and a positive relationship between baPWV and other variables in all subjects, while baPWV values illustrate a strong relationship with SBP, with the coefficient value being 0.60 (*P* < 0.01) (Figure 1).

### ROC curve and its cut-off point

**Figure 2:**
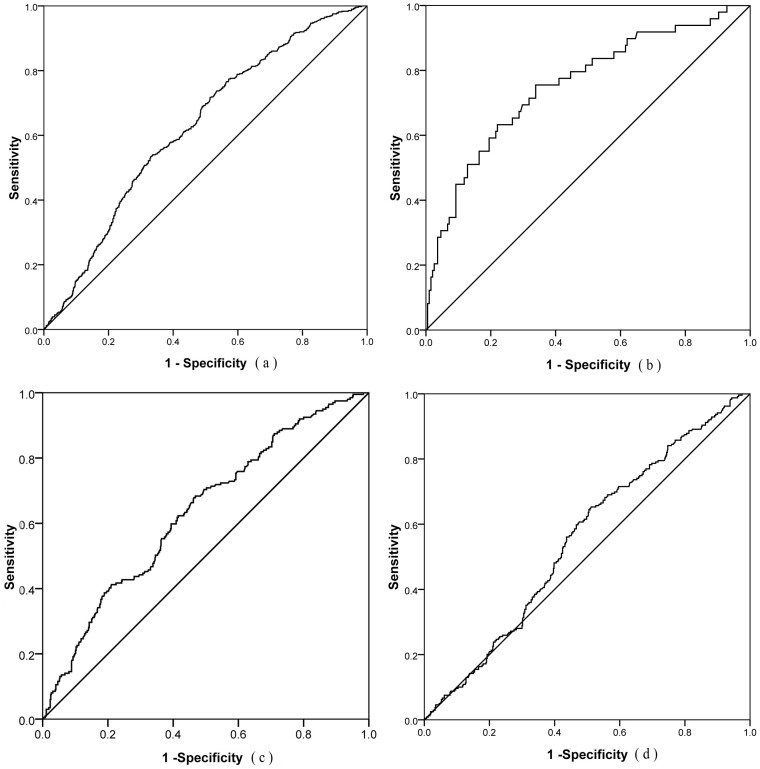
ROC curve and AUC. (a) Total group: AUC = 62.50%, 95%CI of AUC: 60.00%–65.30%, Cut-off point = 1435 cm/sec, Sensitivity = 76.06%, Specificity = 50.43% (Limitation), Youden index = 0.26; (b) 40–50 yrs group: AUC = 75.30%, 95%CI: 67.48%–83.35%; (c) 50–60 yrs group: AUC = 63.35%, 95%CI: 58.96%–67.60%; (d) >60 yrs group: AUC = 55.37%, 95%CI: 51.19%–60.01%.

Figure 2 presents the ROC curves in total group and subgroups (three age ranges). The AUC (95%CI) in total group is 62.50% (60.00%–65.30%) with the appropriate cut-off point being 1435 cm/sec. The AUC in three subgroups (40–50 yrs, 50–60 yrs and over 60 yrs group) are 75.30%, 63.35% and 55.37%, respectively.

Table 4 displays the AUC, cut-off point, sensitivity, specificity, positive predictive value (PPV), negative predictive value (NPV) and Youden index in total group and all three subgroups. The baPWV at cut-off point of 1435 cm/sec has resulted in the highest Youden index in total group, with a corresponding sensitivity being 76.06%, specificity 50.43%, PPV 33.84% and NPV 86.34%. At the same time, its false negative rate is 23.94%.

### Risk factors of MetS in multiple logistic regression model

**Figure 3:**
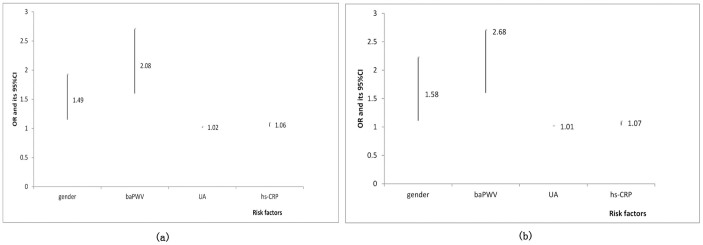
OR value and its 95%CI in different models. (a) Model 1; (b) Model 2.

The MetS status is considered as a dependent variable, the same variables of gender, baPWV, UA and hs-CRP enter into two regression models (Model 1 and Model 2), the regression coefficients in Model 1 are 0.40, 0.73, 0.01 and 0.06, respectively; and the values turn into 0.45 0.73, 0.01 and 0.06, respectively, in Model 2 (Table 5). The Odds Ratio (OR) value (95% CI) of baPWV is the highest in both models, being 2.08 (1.60–2.71) and 2.68 (1.60–2.71), respectively (Figure 3).

When baPWV data are divided into four factions of quartiles and the adjusted variables are same as those in Model 2, the OR and its 95%CI are determined by logistic regression analysis (Table 6). Compared with the readings in lowest baPWV quartile, the adjusted OR (95%CI) of having MetS in baPWV quartiles 2, 3, 4 are 1.60 (1.12–2.27), 1.63 (1.13–2.35), and 2.56 (1.83–3.57) in the total group, respectively.

## Discussion

With the change of living rhythm and dietary style in China, the prevalence of MetS has drastically increased in the last decade throughout the Country. Using the International Diabetes Federation (IDF) definition, Hu XS et al^23^ reported that the prevalence of MetS had been 17.84% in 2005 in Jiangsu Province. In 2010, research carried out by Shao YQ et al^24^ indicated that the prevalence of MetS had been 19.21% in Shanghai area derived from using the same definition. Yet the society lacks a convenient, cost-efficient method to screen for the high-risk MetS individuals in communities.

Brachial-ankle pulse wave velocity (baPWV) is a newly-emerged technique that is capable of evaluating arterial stiffness in a relatively swift and economic way, predicting the cardiovascular risk factors in patients. The subjects with arteriosclerosis share some common pathology and clinical manifestations with subjects suffering MetS; and the existence of a strong association between MetS and arterial stiffness has been demonstrated in many studies^25^,^26^,^27^. In addition, a number of reports have verified that the mechanisms underlying the association include, but not limited to, the common inflammatory pre-cursors and oxidative stress appearance^28^. The simultaneous occurrence of MetS and proinflammatory and oxidative stress markers increases the likelihood of arterial stiffness^29^. Since the baPWV measurements increase before atherosclerosis^30^, we assume that baPWV is likely to be a potential screening tool for MetS.

Our results in this report show that baPWV values correlate with all components of MetS, substantiating the outcomes reported by Zhao WW et al^30^. In general, our data suggest that the prevalence of MetS is 25.65% in the middle-aged and elderly populations in Shanghai area. Although there are some differences in selecting participants with the above studies, our results are consistent with theirs, and the increasing prevalence of MetS in the Chinese population is unquestionably plain.

Thus far, there is not a generally accepted cut-off point of baPWV in screening individuals at risk of MetS. In this study, the discriminatory value of baPWV measurements for MetS has been established in the community population-based study with 1914 subjects. More importantly, we have determined an optimal cut-off point of baPWV (1435 cm/sec), which could be a potential index variable to screen for high risk individuals of MetS in local Chinese populations.

Moreover, besides establishing the useful cut-off point of baPWV, we have calculated baPWV quartiles in logistic model as well. With the increase of baPWV in quartiles, the OR of baPWV for MetS boosts as well, confirming the similar results of an earlier study^31^. The difference between two genders has already been reported in our previous publication^32^.

The ROC curve attained from our results has demonstrated that baPWV may be considered as a screening index to discriminate between those with and without MetS. In total group, the AUC (95%CI) is 62.50% (60.00%–65.30%) and the optimal cut-off point of baPWV for MetS is 1435 cm/sec, which is higher than Yamashina's suggestion^20^, but lower than Lin WY's implication^31^. It is noteworthy that the false negative rate (23.94%) is lower than the previous reports, meaning omission of a diagnostic index is unlikely. In addition, it is interesting to find that the younger a subject's age is, the better ROC efficacy he/she acquires; the higher sensitivity is, the better NPV surfaces. Similarly, the older age is, the higher baPWV appears; the lower specificity is, the lower PPV emerges. This trend is consistent with the results reported by Wang F^33^, who has tried to predict the MetS with BMI. However, our AUC outcome in different subgroups is better than that of the previous report^33^, supporting the notion that baPWV has a better capability for screening MetS than BMI, waist circumference(WC) or waist to hip ratio (WHR).

Analysis of multiple logistic regressions demonstrates that gender, baPWV, UA and hs-CRP are the high risk factors of MetS, consistent with the results of other reports^29^,^34^,^35^. Individuals with higher baPWV are twice more likely to have MetS compared to those with lower baPWV. Hyperuricemia is another significant predictor of MetS^36^, and chronic low-grade inflammation in MetS is an underlying mechanism mediated in part by the pro-inflammatory properties of UA^37^,^38^ and may trigger the elevated baPWV. UA has been found to play two roles in promoting inflammation: as a monosodium urate crystal and as a soluble factor^34^. Inflammation (assessed by hs-CRP) may cling to a possible, underlying relationship between baPWV and MetS^31^,^39^.

It should be pointed out that sensitivity of this screening scheme may be underestimated. Since obesity is an essential diagnostic criterion for MetS and the condition may lead to other metabolic disorders^40^,^41^, a possible limitation exists as we adopt BMI as a surrogate variable for waist circumference in defining people with central obesity^42^. The fact that most Asians rather have normal BMI and high WHR (central obesity) may cause a high false negative rate.

Besides, there are several limitations in the study. Firstly, the study participants consist of middle-aged and elderly individuals, and the AUC is likely underestimated, since the younger age the subgroup members are of, the better ROC efficacy turns up. Secondly, the specificity may not be optimal and most advantageous, and further studies are warranted to verify this. Thirdly, we cannot prove the causality between baPWV and MetS since this is a cross-section study. However, we have found a close association between baPWV and MetS as well as the MetS components, which are also risk factors of cardiovascular diseases. Prospective studies are needed to establish the time sequence in the relationship between UA, hs-CRP, baPWV, and MetS, and to fine tune and improve the screening procedures.

In conclusion, baPWV is a potential index variable to screen high risk individuals of MetS and we would need further work to test the utility as a screening tool. Moreover, a higher baPWV, hs-CRP, UA level, as well as male gender are the independent risk factors for MetS.

## Figures and Tables

**Table 1:** Comparison of demographic characteristics between MetS and Non-MetS group

Variables	MetS group	Non-MetS group	*t*/*χ*^2^	*P*
	mean ± SD	mean ± SD		
Age (years)	60.7 ± 8.9	59.0 ± 8.8	−3.58	0.001
BMI (kg/m^2^)	27.65 ± 2.31	22.95 ± 2.66	−34.37	0.001
Gender [n(%)]				
Male	175(35.64)	444(31.20)	3.28	0.069
Female	316(64.36)	979(68.80)		
Marital status [n(%)]				
Not married	53(10.79)	144(10.12)	0.18	0.671
Married	438(89.21)	1279(89.88)		
Income(RMB per month) [n(%)]				
Low	436(88.80)	1249(87.77)	0.36	0.545
High	55(11.20)	174(12.23)		
Education Level [n(%)]				
Low	409(83.30)	1164(81.80)	0.56	0.453
High	82(16.70)	259(18.20)		
History of diabetes [n(%)]				
Yes	69(14.18)	86(6.11)	31.84	0.001
No	417(85.82)	1329(93.89)		
Smoking [n(%)]				
Yes	372(75.76)	1126(79.13)	2.43	0.119
No	119(24.24)	297(20.87)		
Drinking [n(%)]				
Yes	403(82.08)	1232(86.58)	5.93	0.015
No	88(17.92)	191(13.42)		

Abbreviations: BMI, body mass index;

Marital status: not married refers to both unmarried and divorced individuals;

Income level: high level means greater than or equal to 5000 RMB per month;

Education level: low education level means full time education of less than 12 years, and high level means greater than or equal to 12 years.

**Table 2:** Comparison of biochemical characteristics between MetS and Non-MetS group

Variables	MetS group		Non-MetS group		*t*/*Z*	*P*
	mean	SD	mean	SD		
baPWV (cm/sec)	1677.60	323.14	1554.80	338.52	−6.99	0.001
UA (mg/dL)	350.38	80.88	304.47	76.20	−11.33	0.001
TC (mmol/L)	5.34	0.99	5.17	0.96	−3.19	0.001
TG* (mmol/L)	2.01	(1.56, 2.69)	1.31	(0.97, 1.81)	−10.59	0.001
BNP* (ng/L)	53.00	(29.00, 93.00)	54.00	(32.00, 87.00)	0.02	0.985
Hs-CRP (mg/dL)	2.73	2.83	1.90	3.30	−4.96	0.001
Hcy (mmol/L)	13.02	8.03	12.27	7.34	−1.73	0.060
AI	84.42	12.23	85.68	13.11	1.86	0.063
ABI	1.09	0.08	1.11	0.07	4.87	0.001

*Indicating the variances was not equal between two groups. Data was expressed as median and quartiles value;

Abbreviations: baPWV, brachial-ankle pulse wave velocity; UA, uric acid; TC, total cholesterol; TG, triglyceride; BNP, brain natriuretic peptide; Hs-CRP, hypersensitive C reaction protein; Hcy, homocyteine; AI, augmentation index; ABI, ankle-brachial index.

**Table 3:** Correlation between baPWV and the components of MetS

Variables	*r*	*P*
BMI (kg/m^2^)	0.11	0.001
SBP (mm Hg)	0.60	0.001
TG (mmol/L)	0.14	0.001
HDL-C (mmol/L)	−0.09	0.001
LDL-C (mmol/L)	0.05	0.027
HbA1c (%)	0.19	0.001
Glucose (mmol/L)	0.24	0.001

Abbreviations: BMI, body mass index; SBP, systolic BP; TG, triglyceride; HDL-C, high density lipoprotein cholesterol; LDL-C, low density lipoprotein cholesterol; HbA1c, glycosylated hemoglobin A1c.

**Table 4:** Areas under the ROC curve, cut offs, sensitivity, specificity, PPV, NPV and Youden index of baPWV by age groups

Age	AUC (%) (95%CI)	*P*	Cut-off point	Sensitivity (%)	Specificity (%)	PPV (%)	NPV (%)	Youden index
40–50 (yrs)	75.30 (67.48–83.35)	0.001	1355	75.55	66.29	42.76	89.05	0.42
50–60 (yrs)	63.35 (58.96–67.60)	0.001	1427	68.35	53.28	32.78	83.47	0.22
>60 (yrs)	55.37 (51.19–60.01)	0.018	1680	65.33	48.87	30.03	80.87	0.14
Total	62.50 (60.00–65.30)	0.001	1435	76.06	50.43	33.84	86.34	0.26

Abbreviations: AUC, areas under the ROC curve; PPV, positive predictive value; NPV, negative predictive value.

**Table 5:** Risk factors and parameters of multiple logistic regression model

Variables	ß^d^	SE	Wald	*P*	OR	95%CI
Model1^a^						
Gender	0.40	0.13	9.04	0.001	1.49	(1.15–1.93)
BaPWV^b^	0.73	0.13	30.15	0.001	2.08	(1.60–2.71)
UA	0.01	0.01	92.23	0.001	1.02	(1.01–1.03)
Hs-CRP	0.06	0.01	12.92	0.001	1.06	(1.03–1.10)
Model2^c^						
Gender	0.45	0.17	20.91	0.001	1.58	(1.11–2.23)
BaPWV^b^	0.73	0.13	30.24	0.001	2.68	(1.60–2.71)
UA	0.01	0.00	90.69	0.001	1.01	(1.01–1.02)
Hs-CRP	0.06	0.01	12.89	0.001	1.07	(1.03–1.11)

^a^Adjusted variables in model1: baPWV, gender (male vs. female), educational level, income level, marriage status, UA, Hs-CRP and BNP;

^b^BaPWV abnormal range: ≥1435 cm/sec, normal range, <1435 cm/sec;

^c^Adjusted variables in model2: baPWV, gender, educational level, income level, marriage status, smoking, alcohol drinking, UA, hs-CRP and BNP;

^d^Regression coefficient.

**Table 6:** Odds ratio and 95% CI of baPWV for MetS in regression model

Quartile	Male		Female		Total	
	OR	95%CI	OR	95%CI	OR	95%CI
Q1	1.00	Reference	1.00	Reference	1.00	Reference
Q2	1.40	0.74–2.65	1.66	1.08–2.55	1.60	1.12–2.27
Q3	1.66	0.89–3.10	1.52	0.95–2.41	1.63	1.13–2.35
Q4	2.76	1.55–4.92	2.25	1.48–3.42	2.56	1.83–3.57

Adjusted for baPWV, gender, educational level, income level, marriage status, smoking, alcohol drinking, UA, hs-CRP and BNP; The baPWV was divided into four groups by quartile (<1378, 1378–1569, 1569–1818, ≥1818 cm/sec in men; <1324, 1324–1490, 1490–1738, ≥1738 cm/sec in women; <1336, 1336–1516, 1516–1704, ≥1704 cm/sec in total).
